# Inulin coated plasmonic gold nanoparticles as a tumor-selective tool for cancer therapy†
†Electronic supplementary information (ESI) available. See DOI: 10.1039/c5tb01810b



**DOI:** 10.1039/c5tb01810b

**Published:** 2016-01-04

**Authors:** Anna Li Volsi, Dorleta Jimenez de Aberasturi, Malou Henriksen-Lacey, Gaetano Giammona, Mariano Licciardi, Luis M. Liz-Marzán

**Affiliations:** a Laboratory of Biocompatible Polymers , Department of Scienze e Tecnologie Biologiche Chimiche e Farmaceutiche (STEBICEF) , University of Palermo , Via Archirafi, 32 , 90123 Palermo , Italy . Email: mariano.licciardi@unipa.it ; Fax: +39 091 6100627 ; Tel: +39 091 23891927; b Bionanoplasmonics Laboratory , CIC biomaGUNE , Paseo de Miramón 182 , 20009 Donostia San-Sebastian , Spain . Email: llizmarzan@cicbiomagune.es; c Mediterranean Center for Human Health Advanced Biotechnologies (Med-CHAB) , Palermo , Italy; d Ikerbasque , Basque Foundation for Science , 48013 Bilbao , Spain; e Biomedical Research Networking Center in Bioengineering, Biomaterials, and Nanomedicine (CIBER-BBN) , Paseo de Miramón 182 , 20009 Donostia-San Sebastian , Spain

## Abstract

Polymer coated gold nanospheres are proposed as a tumor selective carrier for the anticancer drug doxorubicin. Thiolated polyethyleneglycol (PEG-SH) and an inulin-amino derivative based copolymer (INU-EDA) were used as stabilizing and coating materials for 40 nm gold nanospheres. The resulting polymer coated gold nanospheres (Au@PEG-INU) showed excellent physicochemical stability and potential stealth like behavior. The system was loaded with doxorubicin (Au@PEG-INU/Doxo) and its cytotoxicity profile was evaluated on human cervical cancer cells (HeLa) and lung cancer cells (A549), as compared to Au@PEG-INU and doxorubicin alone. Cytotoxicity assays showed that the system is able to drastically reduce cell viability upon incubation for 3 days. This result was supported by the ability of Au@PEG-INU/Doxo to be internalized by cancer cells and to release doxorubicin, as assessed by fluorescence microscopy. Finally, a cancer/non cancer cell co-culture model was used to display the advantageous therapeutic effects of the proposed system with respect to doxorubicin alone, thereby demonstrating the ability of Au@PEG-INU/Doxo to preferentially accumulate in tumor cells due to their enhanced metabolism, and to selectively kill target cells.

## Abstract 2

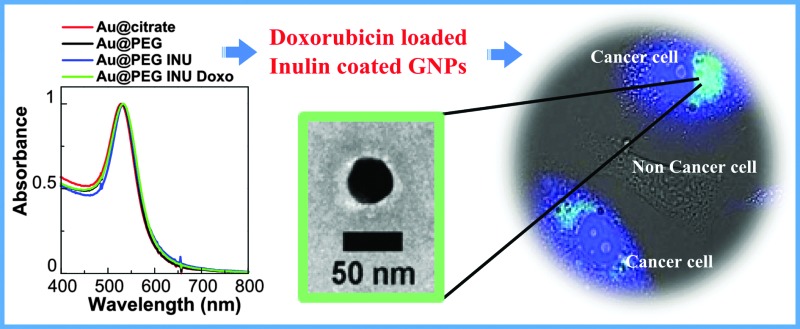
Preferential uptake by cancer cells of PEG-inulin coated gold nanoparticles loaded with the drug doxorubicin.

## Introduction

Cancer is a multifactorial disease caused by genetic and environmental factors that led to over 8 million deaths in 2012.^1^ Conventional approaches used to eradicate tumors include surgery, chemotherapy or radiotherapy, but we have seen over the past decade significant advances in the combination of nanomedicine and drug delivery to produce anti-cancer therapeutics. Nanotechnology is emerging as a promising and powerful tool for medicine with considerable advances in the fields of bioimaging and theranostics.^2^ Importantly, nanotechnology-based therapies for cancer allow reduction of chemotherapeutic dose, improvements in local drug delivery, and reduction in non-specific cytotoxicity. Different approaches have been used for the delivery of cytotoxic cancer drugs, including dendrimers, polymeric particles, polymer coated inorganic nanoparticles and liposomes, the latter being commercialized for the delivery of doxorubicin and other drugs.^3^ Noble metal nanoparticles have also been proposed as nontoxic carriers for drug and gene-delivery applications.^4^ In particular, gold nanoparticles (GNPs) can act as either delivery systems to transport specific therapeutic agents such us small drugs,^5^ or as active elements that additionally provide the possibility to release specifically the bound drug upon irradiation with an appropriate laser beam.^6^ GNPs are versatile agents with unique optical properties (Localized Surface Plasmon Resonances; LSPR) and physicochemical properties that render them appealing for a variety of biomedical applications, including their use in highly sensitive diagnostic assays, thermal ablation and radiotherapy enhancement.^7^ Furthermore, GNPs benefit from easily tunable optical properties as a function of their shape (rods, spheres, triangles, wires *etc.*), size, and composition (*e.g.*, core/shell or alloyed noble metals), with other attractive features such as their high surface-to-volume ratio and the ease of functionalization through thiol bonds.^8^

A wide variety of functionalized GNPs have been prepared by the reduction of AuCl_4_^–^ using organic and inorganic reducing agents, often using citrate as a capping ligand.^9^ However, the reported aggregation of citrate capped GNPs under physiological conditions appear to hinder their *in vivo* applications, often requiring further coatings to ensure high physicochemical stability, biocompatibility and low toxicity. Our group has optimized different polymeric materials that can be exploited for the coating of metallic nanoparticles, including synthetic poly-amino acid derivatives^10^ and inulin based constructs.^11^ Inulin is a natural, biocompatible and biodegradable polysaccharide consisting of linear chains of β-(2-1) fructose units. It exhibits many hydroxyl functional groups that make inulin versatile and easy to functionalize. For example, we recently proposed an amphiphilic inulin based copolymer, capable of self-assembling into micelles, for systemic anticancer drug delivery.^12^ Furthermore, an amino inulin derivative has been used to coat SPIONs yielding stable magnetoplexes by complexation of inulin coated SPIONs with a model duplexed siRNA, for improving oligonucleotide transfection efficiency.^13^ The ability to amino-modulate inulin renders it an attractive candidate for the functionalization of gold nanoparticles, taking advantage of the well documented thiol and amino chemistry at gold surfaces.^14^ We propose here the use of PEG as a pre-stabilizing agent to avoid undesired nanoparticle aggregation,^15,16^ whilst the amino-derived inulin based copolymer is the main biocompatible coating agent for the 40 nm gold nanospheres. This results in a nanosystem that can load doxorubicin while showing stealth like behavior and excellent physicochemical stability but is readily taken up by cancer cells and induces cytotoxicity. We have also shown that larger spherical and rod-shaped gold nanoparticles can be functionalized with the prepared inulin derivative. The ability of the tested drug delivery system to selectively accumulate in malignant but not in normal non-cancerous cells was investigated by using a co-culture cell model.

## Materials and methods

### Materials

Inulin, triethylamine (TEA), ethylendiamine (EDA), bis(4-nitrophenyl)carbonate (BNPC), doxorubicin hydrochloride (DOXO-HCl), gold chloride trihydrate (HAuCl_4_·3H_2_O), sodium citrate tribasic dihydrate, O-[2-(3-mercaptopropionylamino)ethyl]-O′-methylpolyethylene glycol (PEG-SH, *M*_W_ 5000 g mol^–1^) were purchased from Sigma Aldrich. Sephadex G-15 and anhydrous dimethylformamide (a-DMF) were purchased from Fluka (Switzerland). All reagents were of analytic grade, unless otherwise stated. SpectraPor dialysis tubing was purchased from Spectrum Laboratories, Inc. (Italy).

### Synthesis of inulin-2-aminoethyl-carbamate (INU-EDA) polymer

Inulin-2-aminoethyl-carbamate (INU-EDA) was synthetized as previously reported^12^ (for details *cf.* ESI† (SI 1.1)). INU-EDA consists of a linear polysaccharide of glucopyranose end-capped fructose units (β-1,2) (α-d-glucopyranosyl-[β-d-fructofuranosyl](*n*-1)-d-fructofuranoside) bearing primary amine pendant groups. The derivatization degree of pendant primary amine groups (DD_EDA_) is 25 mol% related to inulin monomer units (Fig. S2, ESI†).

### Synthesis and functionalization of nanoparticles

**Fig. 1:**
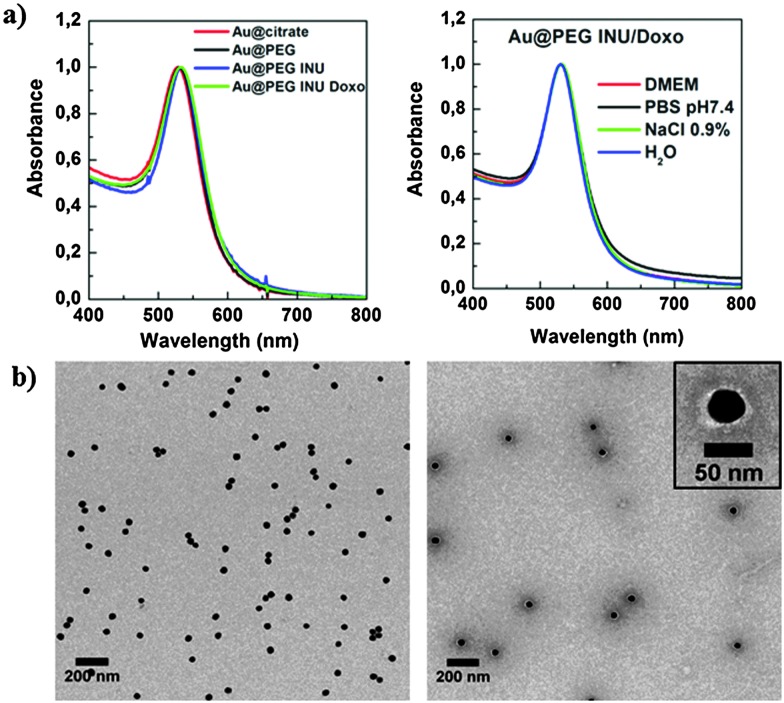
(a) UV-Vis spectra of Au@citrate, Au@PEG, Au@PEG-INU and Au@PEG-INU/Doxo nanoparticles, showing a narrow LSPR band (left) and the stability of Au@PEG-INU/Doxo in different media (right): Milli-Q water, NaCl 0.9%, cell medium (DMEM) and PBS pH 7.4. (b) TEM images of Au@PEG-INU/Doxo without staining (left) and stained with uranyl acetate (right), to distinguish the polymer shell. The inset shows one particle at larger magnification.

Spherical citrate stabilized gold nanoparticles (GNPs) with an average diameter of 40 ± 2.3 nm (Fig. S1, ESI†) were synthesized by seeded growth as recently reported.^17^ As synthesized GNPs featured an LSPR band centered at 529 nm (Fig. 1a). Particles were used without further purification. To an aqueous dispersion of the synthesized GNPs (0.15 mg mL^–1^), PEG-SH solution (381 μL, 6.8 × 10^–6^ M in Milli-Q H_2_O) was added with gentle stirring to obtain a density of PEG-SH molecules per Au nm^2^. The obtained mixture was incubated for 30 min at room temperature. After washing the GNP_S_ by centrifugation at 2760*g* (20 min, 25 °C) to remove unbound PEG-SH, they were dispersed in water.

### Preparation of INU and INU/Doxo functionalized GNPS

A solution of INU-EDA copolymer (952.5 μL, 250 μg mL^–1^) was added to the obtained PEG-SH coated GNPs (Au@PEG), at a ratio of 10 INU-EDA per Au nm^2^. The mixture was incubated for 30 min at room temperature. Coated particles (Au@PEG-INU) were then washed twice by centrifugation at 2760*g* (20 min, 25 °C) and stored at 4 °C. For the preparation of doxorubicin loaded GNPs (Au@PEG-INU/Doxo), 750 μL of an aqueous doxorubicin hydrochloride solution (0.2 mg mL^–1^, 0.34 × 10^2^ mmol), previously treated with TEA (1 μL, 0.7 × 10^–2^ mmol) was added to the INU-EDA solution (952.5 μL, 250 μg mL^–1^).^18^ The obtained mixture was added to an aqueous dispersion of Au@PEG to obtain a Doxo/Au^0^ loading of 20% w/w. After incubation overnight at room temperature the system was washed twice with Milli-Q water to remove excess doxorubicin.

### Determination of drug payload and drug release studies

The amount of drug payload into Au@PEG-INU/Doxo was quantified using two methods: HPLC and UV-vis spectroscopy (for details *cf.* SI 2, ESI†). The amount of doxorubicin loaded per unit mass of Au^0^, quantified by HPLC was 11.8 ± 1.2% (w/w), equivalent to an encapsulation efficiency of 60%. Using the UV-vis spectroscopy method the value was 13 ± 0.5% (w/w), with an encapsulation efficiency of 66%. Drug release over time was measured using dialysis at 37 °C, with aliquots of external medium removed at various time-points (see ESI† for more information).

### Cell culture

All cells (HeLa, A549, MDA.MB.435s, MCF-7, 16-HBE, HDFa and 3T3) were cultured in DMEM media supplemented with 10% fetal bovine serum (FBS) and 1% penicillin–streptomycin (PS). MCF-7 cells were supplemented with 1% sodium pyruvate and 16-HBE and HDFa cells were supplemented with amphotheracin at 250 μg mL^–1^. Cells were maintained in a humidified atmosphere at 37 °C, 5% CO_2_ and passaged using trypsin–EDTA. Cells were obtained from Invitrogen (HDFa), ATCC (A549, MCF-7 and 16-HBE) or were a kind gift from Dr. Charles Lawrie (HeLa), Dr. Ander Izeta (3T3) (both at BioDonostia Institute, San Sebastian, Spain), and Dr. Wolfgang J. Parak (MDA.MB.435s) (CIC biomaGUNE, San Sebastian, Spain). All reagents were purchased from Invitrogen.

### Cell cytotoxicity

Cytotoxicity was studied with HeLa and A549 cell lines which were exposed to Au@PEG-INU/Doxo formulations for 48 h or 72 h. Cell viability was assessed using the MTT or neutral red (NR) assay (for details *cf.* SI 3.1, ESI†).

### Drug delivery studies

The cellular uptake of doxorubicin delivered with Au@PEG-INU NPs was evaluated by fluorescence microscopy analysis. Cells were added to 8-well glass bottomed slides at a concentration of 2 × 10^4^ per well and allowed to adhere overnight. Au@PEG-INU/Doxo and free doxorubicin were added at a final concentration of 40 μg mL^–1^ of Au^0^, corresponding to ∼4 μg mL^–1^ doxorubicin, and cells were incubated for either 4 h, 24 h or 48 h. After each incubation time, cells were washed using PBS and fixed using 4% formaldehyde at room temperature for 20 minutes followed by washing with PBS. Cells were stained first for the nucleus using 4′,6-diamidino-2-phenylindole (DAPI) diluted 1/600 in cell media, and then for the membrane using wheat germ agglutinin-Alexa Fluor® 647 conjugate (WGA647) (1/200 in PBS). Both incubations were carried out at room temperature for 20 min followed by extensive washing. Cells were viewed using a Zeiss AxioVision fluorescence microscope with transmitted light and filters for DAPI, plasma membrane and doxorubicin. To study the tumor selectivity of Au@PEG-INU/Doxo, A549, MDA.MB.435s or MCF-7 cells were co-cultured with normal epithelial cells and/or fibroblasts in glass bottomed 96-well plates (Ibidi; 1.5 × 10^4^ per well) and the passive targeting and uptake of doxorubicin and Au@PEG INU/Doxo were imaged (*cf.* SI 3.2, ESI†).

### Characterization

For the characterization of synthesized INU-EDA polymer, ^1^H NMR spectra were measured using a Bruker Avance II 300 spectrometer operating at 300 MHz (Fig. S3, ESI†). Centrifugations were performed using a Centra MP4R IEC centrifuge. Size exclusion chromatography (SEC) was carried out using a Phenomenex PolySep-GFC-P3999 column (California, USA) connected to a water 2410 refractive index detector. HPLC was carried out using a RP-C_18_ Gemini column (California USA) connected to a refractive index, UV and fluorescence detectors. (NH_4_)_2_HPO_4_/acetonitrile 68 : 32 (v/v) solution was used as eluent at 25 °C with a flux of 0.8 mL min^–1^. To characterize nanoparticles, transmission electron microscopy (TEM) images were collected with a JEOL JEM-1400PLUS microscope operating at 120 kV, using carbon coated 400 square mesh copper grids. Negative staining using uranyl acetate was used to image the polymer shell. UV-Vis extinction spectra were recorded using an Agilent 8453 UV-Vis diode-array spectrophotometer. Dynamic light scattering (DLS) and zeta-potential measurements were performed at 25 °C using a Malvern ZetasizerNanoZS instrument, fitted with a 532 nm laser at a fixed scattering angle of 173°. Qualitative elemental composition of freeze-dried Au@PEG-INU/Doxo was determined using an environmental scanning electron microscope (ESEM) Philips XL30. Samples were casted on a double sided adhesive tape previously applied on a stainless steel stub prior to elemental analysis. Fluorescence imaging was performed with an Axio Cell Observer (Zeiss) with LED and HXP lamp source.

## Results and discussion

### Preparation and characterization of PEG-SH and INU-EDA coated GNPs

Polymer coated gold nanoparticles were obtained through a double coating process. GNPs stabilized in citrate buffer (Au@citrate) were first capped with the pre-stabilizing agent PEG-SH and the amino derivative of inulin, INU-EDA, was subsequently added as the main biocompatible polymeric coating agent. Due to the high affinity of gold for the thiol and amino groups of PEG-SH and INU-EDA respectively, a hydrophilic polymeric coating was obtained that could permanently stabilize GNPs in aqueous media. This procedure was also suitable for different sizes and shapes of gold nanoparticles. As shown in Fig. 1, the redshift of the LSPR band without broadening of 40 nm gold nanoparticles and the well separated particles observed in TEM images confirmed the absence of aggregation, as well as the preservation of particle size after functionalization. The same was observed for 80 nm particles and rod-shaped gold nanoparticles coated with INU-EDA (Fig. S4, ESI†).The stability of the final doxorubicin containing NPs in cell culture media (DMEM), PBS, salt solutions and water was tested. As also shown in Fig. 1a, there was no significant change in the absorbance spectrum of the Au@PEG-INU/Doxo particles. This was confirmed by DLS and zeta potential measurements (Table S1, ESI†).The organic stabilizing layer could be clearly seen when the GNPs were negatively stained (Fig. 1b, inset).

The occurrence of polymer coating on the GNPs surface was further confirmed by qualitative energy dispersion X-ray elemental analysis (Fig. S5, ESI†), which showed the characteristic peaks of carbon, oxygen and nitrogen, in agreement with the presence of a polymeric shell with a minimum thickness of 5 nm, which is the average resolution depth of the instrument. DLS measurements supported the results obtained *via* TEM, yielding an average hydrodynamic diameter of 60 nm and a polydispersity index of 0.19 (Table S2, ESI†). Zeta potential measurements (Table S2, ESI†) resulted in values of +20.5 mV for Au@PEG-INU, in contrast with –29.1 mV for Au@citrate NPs, which confirmed successful association of the PEG-INU polymeric coating. Doxorubicin loaded Au@PEG-INU NPs were further characterized to determine the efficacy of drug loading. A benefit of using doxorubicin is that its natural fluorescent properties can be exploited to evaluate drug loading and release, as well as further fluorescence microscopy experiments in cells with no need for prior labeling. Drug loading was carried out by adding aqueous INU-EDA and doxorubicin hydrochloride solutions, previously treated with TEA, to the dispersion of Au@PEG. A molar excess of TEA was added to the drug solution to obtain doxorubicin-free base, which in spite of a lower solubility in water, provides higher affinity for the hydrophobic gold core. The driving forces that allow encapsulation of doxorubicin in Au@PEG INU nanoparticles are most likely physical interactions including hydrogen bonding between the sugar ring of doxorubicin molecules and the polysaccharide backbone of INU-EDA, as well as hydrophobic interactions between the hydrophobic drug domains and the gold core. The amount of loaded drug, as evaluated by HPLC analysis, was found to be 12% w/w, with an encapsulation efficacy of approximately 60% (Table S2, ESI†). This value was confirmed by UV spectroscopy of the supernatant recovered during preparation of the nanoparticles, by evaluating the difference between the total amount of drug used during preparation and the drug in the supernatant. DLS measurements carried out on Au@PEG-INU/Doxo, as well as TEM analysis, showed no significant variation in size and zeta-potential as compared to the unloaded system Au@PEG-INU (Table S2, ESI†).

To better understand the usefulness of INU-EDA as a coating agent, we compared Au@PEG-INU/Doxo to its analogue Au@PEG/Doxo (not functionalized with INU-EDA copolymer). The latter was prepared using the same procedure described in the methods section but without addition of INU-EDA copolymer during the drug loading procedure. In particular, the capability of drug loading and the stability in PBS pH 7.4 were investigated for both systems (Table S2 and Fig. S6, ESI†). The amount of doxorubicin loaded into Au@PEG/NPs was calculated to be 5 ± 0.8% w/w with an encapsulation efficiency of 29%. Compared to their INU containing counterparts (60% encapsulation efficiency), these results clearly prove that the presence of INU-EDA on the NP surface largely improved drug loading, thus supporting the proposed interaction between the polysaccharide backbone of INU-EDA and doxorubicin. The stability studies also showed that INU-EDA enhanced the physicochemical stability of the colloid when stored in PBS (pH 7.4, 25 °C), which was also verified by UV-Vis spectroscopy (Fig. S6, ESI†).

### Doxorubicin release studies

**Fig. 2:**
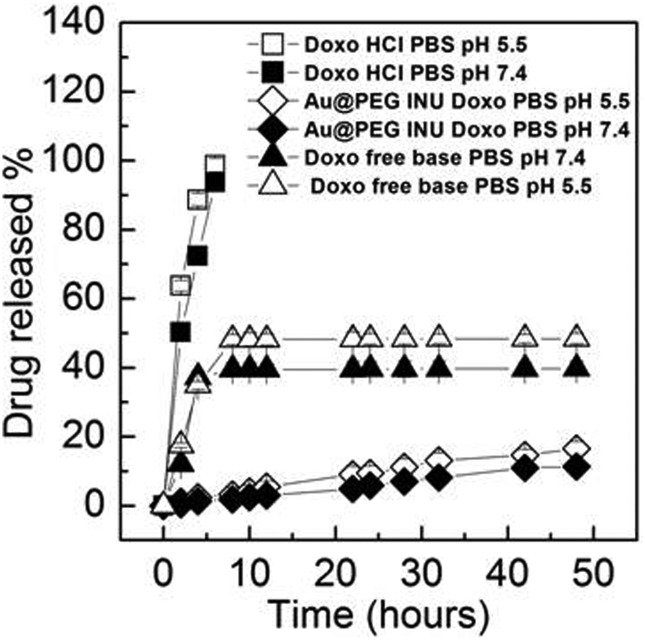
Percentage doxorubicin release from Au@PEG-INU/Doxo NPs over time, when stored in PBS solutions at pH 7.4 and pH 5.5. Doxorubicin not associated with NPs, both in the free base (–NH_2_) and hydrochloride forms, were used to determine normal doxorubicin release during dialysis.

Drug release experiments were carried out at both pH 7.4 and pH 5.5, simulating the difference between endosomal and lysosomal environments, respectively (Fig. 2). The amount of doxorubicin released from Au@PEG-INU NPs was plotted as a function of the incubation time and compared to the free drug (the latter used as hydrochloride and free base). Au@PEG-INU NPs proved capable of releasing doxorubicin for a prolonged period of time and without any initial “burst effect”, for both pH values. For example, after 48 h approximately 13% and 15% of doxorubicin initially loaded in the system were slowly released in PBS at pH 7.4 and pH 5.5, respectively, suggesting that a slow drug release rate could be obtained even after reaching the targeted site of action.

Interestingly, the results suggest that the pH of the medium does not substantially affect the release profile. Taking into consideration that both at pH 7.4 and at pH 5.5 the protonated forms of the amino groups of INU-EDA and of doxorubicin (–NH_3_^+^) are prevalent, (p*K*_a_ of the Doxo amino group is 8.46), a similar trend in drug release at both pH values is expected, most likely due to a slow desorption of the drug from the nanoparticle core.

### *In vitro* assays

**Fig. 3:**
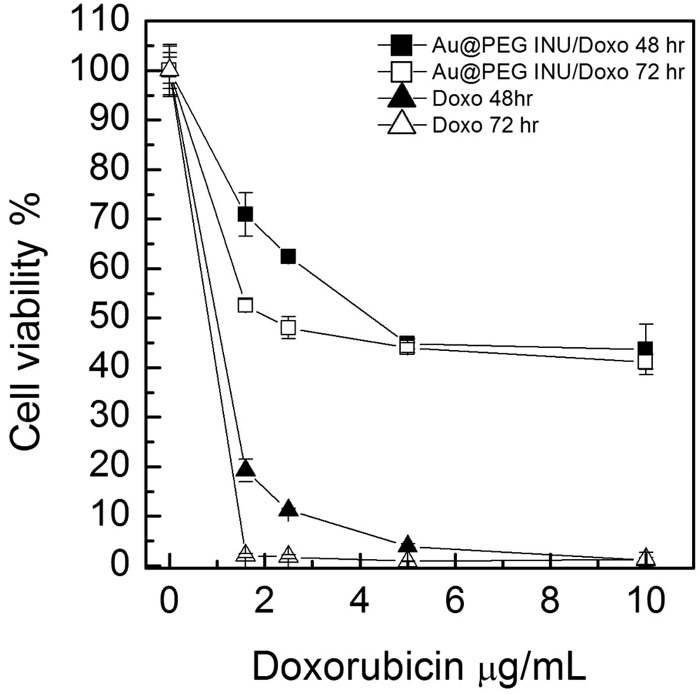
Cell viability of human cervical cancer cells (HeLa) after exposure to free doxorubicin hydrochloride or Au@PEG-INU/Doxo NPs, for either 48 h or 72 h.

**Fig. 4:**
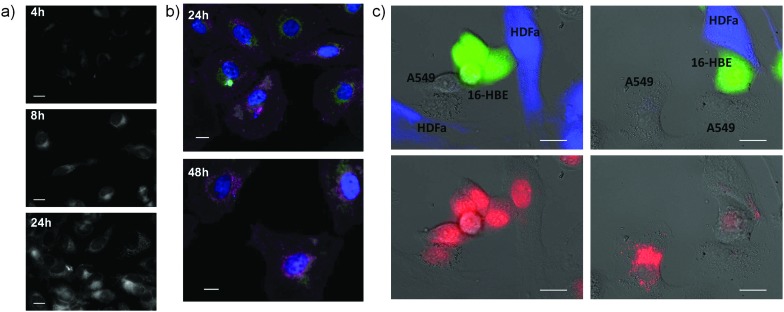
Fluorescence imaging of doxorubicin uptake when delivered *via* Au@PEG-INU NPs to cancer cells. (a) Increasing intracellular presence of doxorubicin in HeLa cells at 4 h, 8 h and 24 h. (b) Multistained HeLa cells showing doxorubicin (green), nuclear (blue) and membrane (pink) staining. The decreasing amount of cells is clear when longer incubation times are used. (c) Co-culture of A549 lung cancer cells combined with normal 16-HBE epithelial and HDF fibroblast cells. 16-HBE and HDF cells were pre-stained with CMFDA (green) and CMF_2_HC (blue) respectively. Doxorubicin alone diffuses equally in all three cells (left panel) whereas preferential uptake of Au@PEG INU/Doxo by A549 cells is visible (right panel). Cell tracker staining alone is shown in top images and doxorubicin fluorescence in bottom images for clarity. Scale bar: 20 μm.

The cytotoxic ability of Au@PEG-INU/Doxo NPs was tested with various human cancer cell lines. HeLa and A549 cells were exposed to Au@PEG-INU NPs, with and without doxorubicin, as well as to free doxorubicin, for either 48 h or 72 h (Fig. 3, Fig. S7, ESI†). In the case of Au@PEG-INU NPs, no toxicity was noted over the range of concentrations and time points tested (Fig. S8, ESI†). We were interested to see whether the incorporation of doxorubicin in Au@PEG-INU NPs resulted in a slower onset of cell death, due to the slow release action of the system. Using the same MTT assay, which measures the metabolic activity of cells, we noted that after 72 h incubation of NPs with cells, such high levels of cytotoxicity were not achieved. Cell death was both time and concentration dependent with little difference between HeLa or A549 cells. The IC_50_ values for free doxorubicin and for the Au@PEG INU/Doxo system with HeLa cells are reported in Table 1 (for IC_50_ with the A549 cell line *cf.* Table S3, ESI†). As expected, free doxorubicin was highly toxic. The internalization of Au@PEG-INU/Doxo into cancer cells was analyzed by fluorescence microscopy using the same two cell lines, HeLa (Fig. 4) and A549 (Fig. S9, ESI†). Images obtained at 4 h after exposure of cells to Au@PEG-INU/Doxo NPs showed that doxorubicin was present in the cytoplasm of the cells (nuclear staining highlights the typical moon-shape of cytoplasmic staining). In contrast, exposure of cells to free doxorubicin led to rapid (<4 h) staining of the nucleus and important loss of cells from the glass substrate between the 8 h and 24 h time-points (results not shown), clearly illustrating the difference compared to the slow release of doxorubicin from Au@PEG-INU NPs.

**Table 1:** IC_50_ of Au@PEG INU/Doxo and free doxorubicin on HeLa cells after 48 h and 72 h of incubation

Sample	IC48h50 (μM)	IC72h50 (μM)
Free doxorubicin	0.88	0.4
Au@PEG INU Doxo	3.8	1.9

We additionally studied the ability of Au@PEG-INU/Doxo to preferentially accumulate in cancer rather than non-cancer cells, thereby achieving a selective drug accumulation into cancer cells and avoid damage to surrounding healthy cells and systemic toxicity in general, often produced by conventional chemotherapeutics.^19^ In order to achieve this we used various co-culture models composed of a cancerous cell combined with a healthy epithelial cell line and/or fibroblast.^20^ After a short incubation time of 90 min (so as to clearly see the differences between cancer and non-cancer cells lines), cells were thoroughly washed. Shown in Fig. 4 is the preferential uptake of doxorubicin by A549 cells when delivered combined with Au@PEG-INU. The presence of doxorubicin in the cytoplasm is only seen in the cancerous A549 cells, supporting the well-known increased metabolic rate of cancer cells as compared to non-cancer cells.^21,22^ This result is also seen in various double co-cultures including MCF-7/HDFa or A549/3T3 (Fig. S9 and S10, ESI†). The presence of doxorubicin, when delivered without a NP carrier, in non-cancer cells at such short time-points could explain doxorubicin's toxicity towards normal tissues and cells. As a matter of fact, this first-line anticancer agent is effective against a wide spectrum of neoplasms, but is also responsible for several cumulative dose dependent adverse effects, including cardiomyopathy, typhilitis, and acute myelotoxicity.^23^

## Conclusions

We report the synthesis of 40 nm gold nanospheres including a double coating process with PEG–thiol as a stabilizer and a novel amino derivative of inulin (INU-EDA). The presence of thiol and amine groups allowed us to easily functionalize GNPs' surface, leading to a stable polymer coating with a total hydrodynamic diameter of about 60 nm. The use of biocompatible and biodegradable INU-EDA as a further coating copolymer was found to lead to two important advantages: an enhancement of the amount of loaded drug and an improvement in the physical stability of the system in physiological mimicking media. Moreover, it has been proven that the procedure followed to coat particles with INU-EDA is suitable for other sizes and shapes of gold nanoparticles providing possibilities for further applications such as light-controlled drug release. The Au@PEG-INU/Doxo system was able to incorporate 12% w/w doxorubicin, *i.e.* at least twice the reported amount measured in the inulin-free system. The physicochemical features of Au@PEG-INU NPs, with or without doxorubicin, remained unchanged after two weeks in buffer solution, thus showing stability. The polymeric shell allowed stabilization of the gold core in aqueous media and resulted in a slow release of doxorubicin within the therapeutic range. Au@PEG-INU/Doxo NPs showed good anticancer activity in human cancer cell lines (HeLa, A549, MCF-7 and MDA.MB.435s cells). Furthermore, the intracellular location of doxorubicin in the cytoplasm supported the slow release of the drug, and there was preferential accumulation of doxorubicin into cancer cells rather than non-cancer cells when delivered with the NP. We have thus shown differences in particle uptake between two cancer cell lines and a co-cultured fibroblast cell. These results offer advantageous therapeutic effects regarding increased anticancer activity through the tumor mass and reduced non-specific cytotoxicity to surrounding healthy tissues, supporting the possible selection of Au@PEG-INU/Doxo as a promising candidate for future *in vivo* studies.
