# Supplementary material for: Inulin coated plasmonic gold nanoparticles as a tumor-selective tool for cancer therapy
Source: J Mater Chem B. 2016 Jan 4;4(6):1150–5. doi: 10.1039/c5tb01810b (PMC5317210; doi:10.1039/c5tb01810b)
Supplement: Supplementary file 1 [file TB-004-C5TB01810B-s001.pdf]

Electronic Supplementary Material (ESI) for Journal of Materials Chemistry B.
This journal is © The Royal Society of Chemistry 2016

Inulin Coated Plasmonic Gold Nanoparticles as a Tumor-Selective Tool for Cancer Therapy

Anna Li Volsi[1], Dorleta Jimenez de Aberasturi[2], Malou Henriksen-Lacey[2], Gaetano Giammona[1,3], Mariano Licciardi[1,3]*, Luis M. Liz-Marzán[2,4,5]*

[1]Department of Scienze e Tecnologie Biologiche Chimiche e Farmaceutiche (STEBICEF), University of Palermo, Italy.

[2]Bionanoplasmonics Laboratory, CIC BiomaGUNE, Paseo de Miramón 182, 20009 Donostia San-Sebastián, Spain.

[3]Mediterranean Center for Human Health Advanced Biotechnologies (Med-CHAB), Palermo, Italy.

[4]Ikerbasque, Basque Foundation for Science, 48013 Bilbao, Spain.

[5]Biomedical Research Networking Center in Bioengineering, Biomaterials, and Nanomedicine (CIBER-BBN), Paseo de Miramón 182 , 20009 Donostia- San Sebastián, Spain.

*Authors to whom correspondence should be addressed:

- mariano.licciardi@unipa.it, Laboratory of Biocompatible Polymers, Department of Scienze e TecnologieBiologiche, Chimiche e Farmaceutiche (STEBICEF), University of Palermo, Via Archirafi, 32 90123 Palermo, Italy. Tel.: +39 09123891927; fax: +39 091 6100627.
- llizmarzan@cicbiomagune.es, Bionanoplasmonics Laboratory, CIC BiomaGUNE, Paseo de Miramón 182, 20009 Donostia San-Sebastián, Spain.

SUPPLEMENTARY INFORMATION

SI 1. Nanoparticle and polymer characterization

SI 1.1 Synthesis of INU-EDA copolymer

Figure S1. TEM image of spherical citrate stabilized 40 nm gold nanoparticles (Au@citrate)

Figure S2. Scheme of the synthesis of INU-EDA copolymer

Figure S3. ^{1}HNMR spectrum of INU-EDA copolymer

Figure S4. TEM images and Vis-NIR spectra of 80 nm and rod-shaped gold nanoparticles.

Figure S5. Energy dispersive X-ray analysis of the surface of Au@PEG-INU.

Table S1. DLS data and zeta potential values of Au@PEG-INU/Doxo in Milli-Q water, NaCl 0.9% (w/v), PBS pH 7.4 and in cell medium (DMEM).

Table S2. DLS data, zeta potential values and drug loading % of Au@citrate, Au@PEG INU and Au@PEG INU/Doxo

Figure S6. Stability studies of Au@PEG Doxo and Au@PEG INU Doxo in PBS at pH 7.4

SI 2. Determination of drug payload into Au@PEG-INU/Doxo

SI 3. Cell studies and exposure to nanoparticles

SI 3.1. Cytotoxicity assay on human cervical cancer cells (HeLa) and on human lung cancer cells (A549)

SI 3.2 Cell drug uptake studies: *Co-culture of cancer cells with fibroblasts*

Figure S7. Cell viability of A549 cells

Figure S8. Cell viability of A549 and HeLa cells after exposure to Au@PEG INU NPs.

Table S3. IC_{50} values of Au@PEG INU/Doxo and free doxorubicin for A549 cells after 48 and 72 h of incubation.

Figure S9. Uptake of Doxorubicin in A549 lung cancer cells and co-culture of human breast cancer cells with fibroblasts.

Figure S10. Co-cultures of A549 and MDA.MB.435 cancerous cells with 3T3 fibroblasts.

SI 1. Nanoparticle and polymer characterization

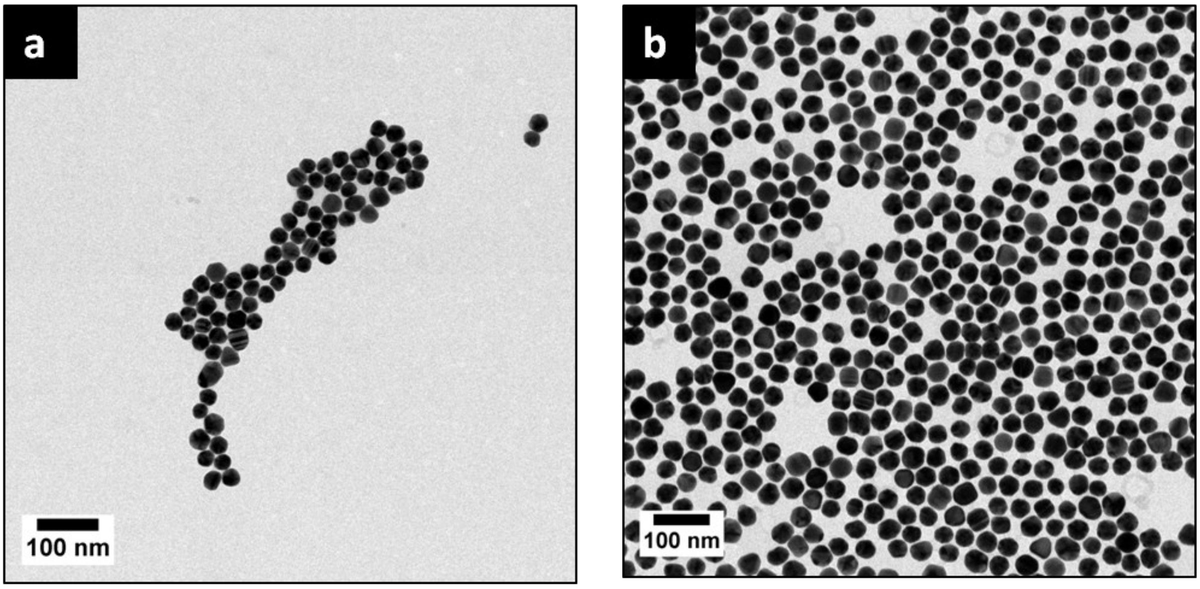

Figure S1. TEM image of a) Au@citrate NPs and b) Inulin coated NPs (Au@PEG-INU).

SI 1.1 Synthesis of INU-EDA copolymer

Inulin (250 mg), dissolved in anhydrous N,N-Dimethylformamide (a-DMF) (4 mL), was activated by reacting with Bis 4-nitrophenyl carbonate (BNPC) (236 mg in 1mL of a-DMF) in a CEM Discover Microwave Reactor for a 1 h with a power of 25 W. The reaction temperature was monitored and maintained at 50 °C by cooling with external compressed air. Then the reaction mixture was added drop-wise to an ethylenediamine (EDA) solution in a-DMF. The reaction mixture was kept under constant stirring for 1 h at 25 °C. After this the obtained product was precipitated in a diethyl ether–dichloromethane mixture (2:1 v/v) and the solid residue recovered and washed with acetone to remove the excess of unreacted EDA and BNPC. Then, the solid product was purified by gel permeation chromatography using Sephadex G-15 as the separating gel. Typically a yield of 95-100 % was obtained. The obtained copolymer INU-EDA **(Figure S2)** was characterized by ^{1}H NMR (300 MHz, D_2O) and spectroscopic data were in agreement with attributed structure: δ 2.74-3.15 ($4H_{EDA}$, -NH-**CH_2-CH_2**-NH_2), 3.55- 4.10 (5 H_{INU}, -**CH_2**-OH; -**CH**-CH_2-OH; -C-**CH_2**-O-), 3.92-4,30 ($2H_{INU}$, -C-**CH**-OH; -**CH**-OH) **(Figure S3).**

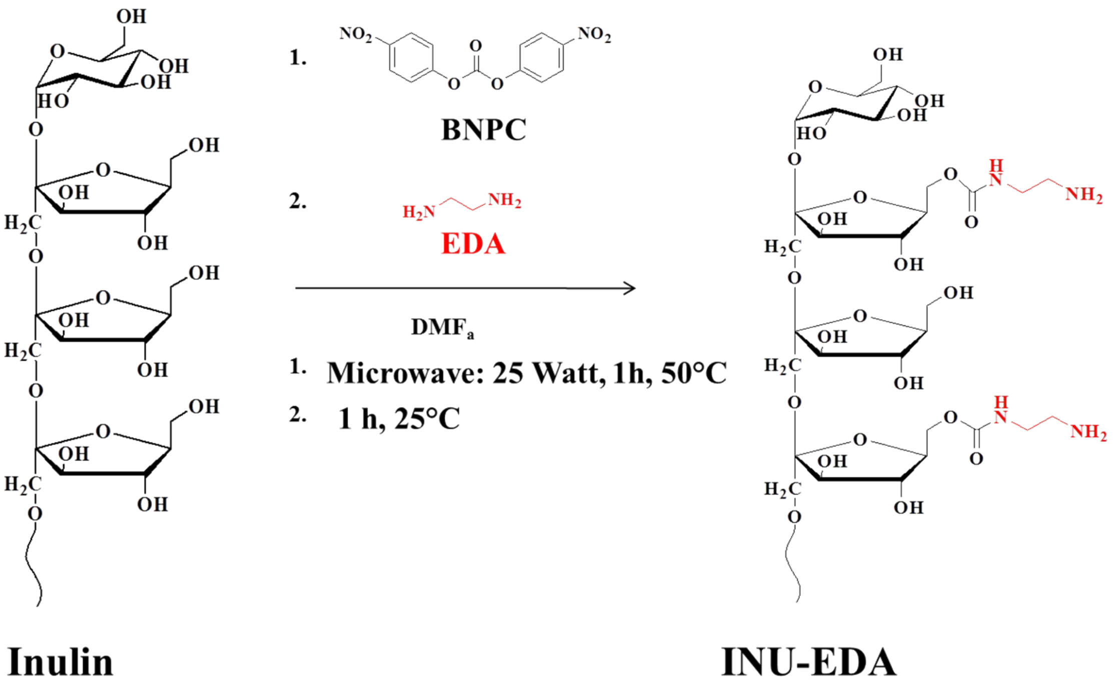

Figure S2. Scheme of the synthesis of INU-EDA copolymer.

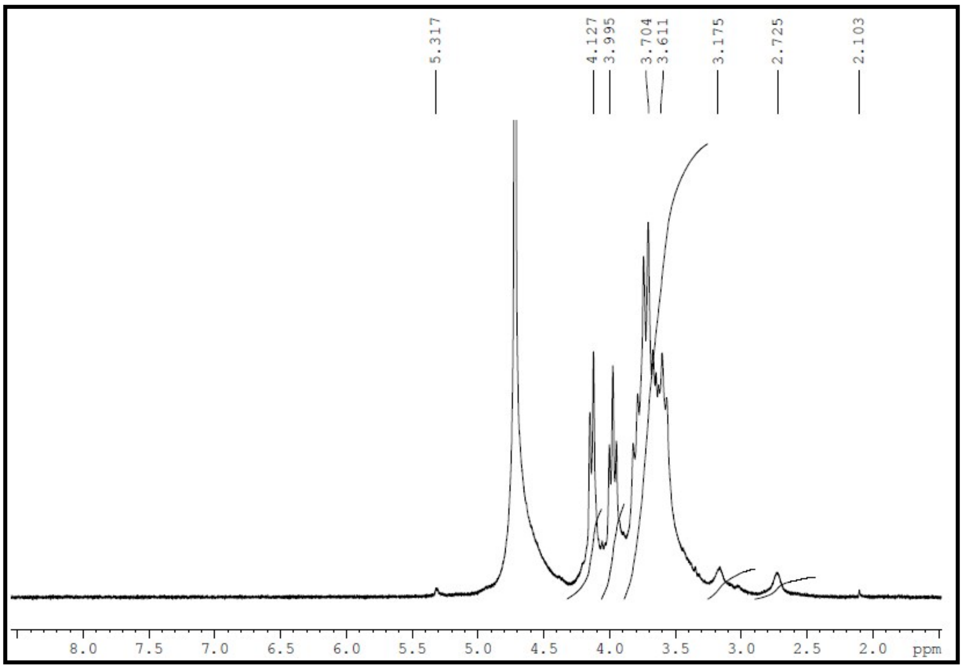

Figure S3. ^{1}H NMR spectrum (Bruker Avance II 300, at 300 MHz, D_2O) of INU-EDA copolymer. Spectroscopic data are in agreement with attributed structure: δ 2.72-3.17 ($4H_{EDA}$, -NH-CH_2-CH_2-NH_2), 3.611- 3.704 (5 H_{INU}, -CH_2-OH; -CH-CH_2-OH; -C-CH_2-O-), 3.99-4.12 ($2H_{INU}$, -C-CH-OH; -CH-OH).

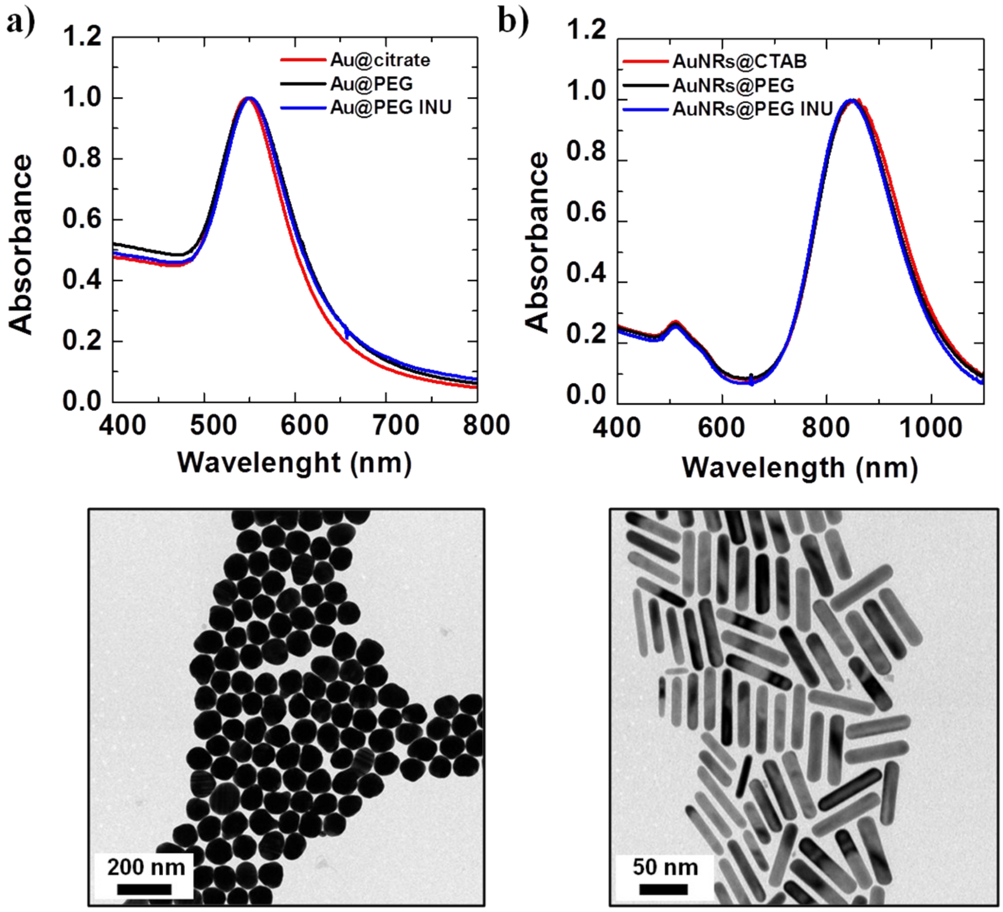

Figure S4. Examples of gold nanoparticles with different size and shape coated with INU-EDA. Vis-NIR spectra of a) 80 nm citrate nanoparticles and b) CTAB stabilized rod-shaped nanoparticles (red), coated with PEG (black) and PEG/INU (blue). TEM images of a) and b) with INU-EDA functionalization.

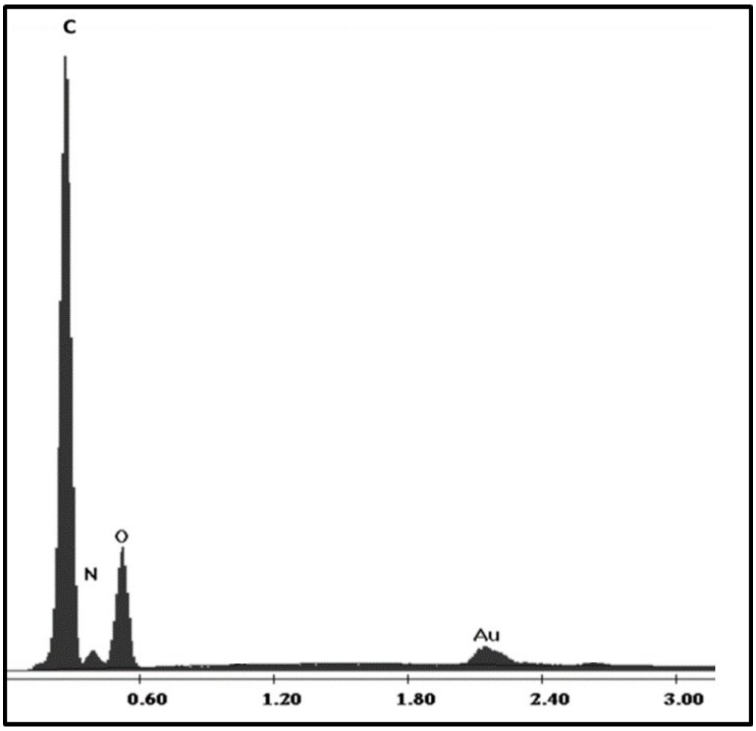

Figure S5. Energy dispersion X-ray chemical analysis of the surface of Au@PEG-INU.

Table S1. DLS data and zeta potential values of Au@PEG INU-Doxo (0.1 mg/mL of Au^0) in Milli-Q water, NaCl 0.9% (w/v), PBS pH 7.4, and in cell culture medium (DMEM).

Au@PEG-INU/Doxo in	Z average (nm)	PDI	Z potential (mV)
H_2O Milli-Q	58.7	0.21	14.3
NaCl 0.9%	48.7	0.1	-0.1
PBS pH 7.4	57.7	0.13	-0.8
DMEM (cell medium)	55.5	0.11	-3.2

Table S2: DLS data, zeta potential values and drug loading % of Au@citrate, Au@PEG-INU and Au@PEG-INU/Doxo samples in aqueous media (0.1 mg Au^0/mL).

Sample	Z average (nm)	PDI	Zeta Potential (mV)	Drug Loading % (w/w) evaluated by HPLC method	Encapsulation efficiency % (w/w)
Au@citrate	45.1	0.2	-29.1	-	-
Au@PEG	54.3	0.18	-15.2	-	-
Au@PEG-INU	61.6	0.19	20.5	-	-
Au@PEG-INU/Doxo	58.7	0.21	14.3	11.8±1.2	59.9
Au@PEG/Doxo	-	-	-	5 ± 0.8	29

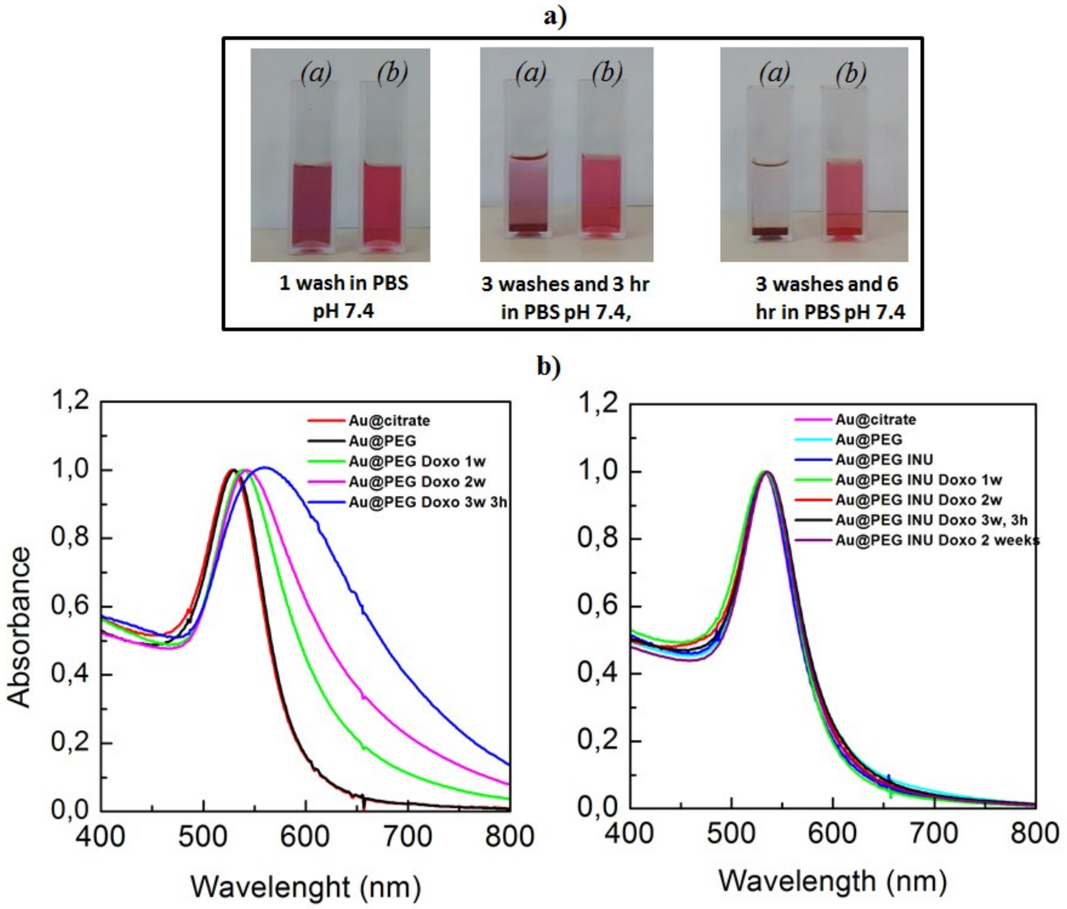

Figure S6. Stability studies of Au@PEGDoxo and Au@PEG INU Doxo in PBS at pH 7.4. a) Photos of Au@PEG/Doxo*(a)* and Au@PEG-INU/Doxo*(b)* after 1 wash in PBS pH 7.4, after 3 washes and 3 hours in PBS pH 7.4 at 25°C and after 3 washes and 6 hours in PBS pH 7.4 at 25ºC. b) UV-Vis spectra of Au@PEG/Doxo (left) and Au@PEG-INU/Doxo (right) in PBS pH 7.4 after different washes (w).

SI 2. Determination of drug payload into Au@PEG INU/Doxo

The amount of drug payload into Au@PEG-INU/Doxo was measured by using two methods: HPLC and UV-Vis spectroscopy. In the first method, a precise amount of Au@PEG INU/Doxo was dispersed in Milli-Q water pH 4.5 to extract the loaded drug. After 30 min of incubation under stirring, the dispersion was centrifuged (5000 rpm, 15 min) and the supernatant freeze dried. The freeze dried supernatant was solubilized in the HPLC solvent ($(NH_4)_2HPO_4$ 0.4 M / Acetonitrile 68:32 v/v), filtered on a cellulose regenerated filter, cut off 0.2 μm, and injected in a C_{18}Gemini HPLC column. The content of drug loaded into the system was calculated by using a calibration curve obtained for serially diluted concentrations of doxorubicin in the eluent and expressed as the amount of loaded doxorubicin per unit mass of Au^0, which resulted to be 11.8±1.2 % (w/w), equivalent to an encapsulation efficiency of 59.9 %. In the second method, an appropriate amount of Au@PEG-INU/Doxo was washed several times with water and the dispersion centrifuged (5000 rpm. 20 min) to remove the excess of doxorubicin. The supernatants were collected and analyzed by UV-Vis spectroscopy measuring the absorbance at 543 nm to determine the amount of excess Doxorubicin not loaded into Au@PEG INU/Doxo system. A calibration curve was obtained for serially diluted concentrations of doxorubicin in distilled water. The content of drug loaded into the system was calculated as a difference from the total amount of doxorubicin used during the preparation of Au@PEG-INU/Doxo and expressed as the amount of loaded doxorubicin per unit mass of Au^0, and resulted to be 13±0.5 % (w/w), resulting in an encapsulation efficiency of 66%.

SI 3. Cell studies and nanoparticle exposure

SI 3.1 Cytotoxicity assay on human cervical cancer cells (HeLa) and on human lung cancer cells (A549)

The cytotoxicity of Au@PEG INU, Au@PEG INU/Doxo and doxorubicin alone was assessed by the MTT assay on human cervical cancer cells (HeLa) and human lung cancer cells (A549). Cells were detached from growth flasks, counted and plated in 96-well tissue culture plates at a concentration of 2 x 10^5 cells/mL, 100 µL/well. Upon attachment by incubation overnight, the medium was replaced with 200 µL/well of fresh cell medium containing empty Au@PEG INU and Au@PEG INU/Doxo at a concentration per well equal to 100, 50, 25 and 16 µg/mL of Au^0, corresponding to 10, 5, 2.5 and 1.6 µg/mL of doxorubicin per well. After 48 or 72 h, supernatants were removed and MTT assays conducted. A 1/20 dilution of MTT reagent in media was added to the wells (100 µl/well) and incubated at 37 ºC for 1 h. Cells were washed twice and 100 µL/well of DMSO was added. Absorbance at 550 nm was read using a plate reader. Doxorubicin hydrochloride solutions at the same concentrations and cell media were used as positive and negative controls respectively. In some experiments the neutral red assay was used. This differed from the MTT assay in that a solution of neutral red in media (0.1mg/ml) was added to cells instead of the MTT solution. Cells were left for 1hr, followed by removal of the solution and addition of a "stop" solution (ethanol, acetic acid and water) to lyse cells. Absorbance at 550 nm was read using a plate reader.

SI 3.2 Cell drug uptake studies

Co-culture of cancer cells with fibroblasts

In order to study the tumor selectivity of Au@PEG INU/Doxo, we set up various co-cultures representing breast or lung cancer tissues. Specifically, A549 bronchial adenocarcinoma cells were co-cultured with normal 16-HBE bronchial epithellial cells and HDFa fibroblasts, or with 3T3 fibroblasts. MDA.MB.435 melanoma metastases cells were cultured with 3T3 fibroblasts, and MCF-7 breast epithelial adenocarcinoma cells with HDFa fibroblasts. All cell co-cultures were set up in Ibidi 96-well µ-plates with an optical bottom. For 3T3 cell containing co-cultures, A549 or MDA.MB.435s cells were plated at a density of 1 x 10^5/mL, 200 µL/well and allowed to adhere. Cells were stained using Cell tracker CMF2HC dye

(Invitrogen) at a final concentration of 0.1 mM for 1h at 37°C. Cells were thoroughly rinsed and allowed to stabilize for a few hours or overnight. 3T3 fibroblasts were harvested and plated in wells containing A549 or MDA.MB.435s cells at a final concentration of 1 x 10^5/ml, 200 µL/ well and allowed to adhere. For remaining co-cultures 16-HBE and HDFa cells were plated in 24-well plates at 1.4 x 10^4/well and allowed to grow for 48h. 16-HBE cells were stained with CMFDA (1/1000, 30 min, 37°C; Invitrogen) and HDFa with CMF_2HC (1/100, 1h, 37°C), followed by at least 2 washes with warmed media. Cells were left for an hour to stabilize and were then uplifted using Trypsin-EDTA and counted. Co-cultures of A549 with 16-HBE (green) and HDFa (blue), and MCF-7 with HDFa (blue) were made in Ibidi 96-well µ-plates at a final concentration of 1.5 x 10^4/well. The following day, media was replaced with solutions of doxorubicin or Au@PEG-INU/Doxo, at a final concentration of 40 µg/Au^0 corresponding to ~ 4 µg/mL doxorubicin. Cells were imaged from 30 min onwards and based on uptake, cells were thoroughly washed 1.5 h after addition of doxorubicin, with or without NPs. Cells were immediately imaged using a Cell observer Zeiss microscope with temperature and CO_2 control, using a Dapi filter to image the Cell tracker and a Rhodamine filter to image doxorubicin.

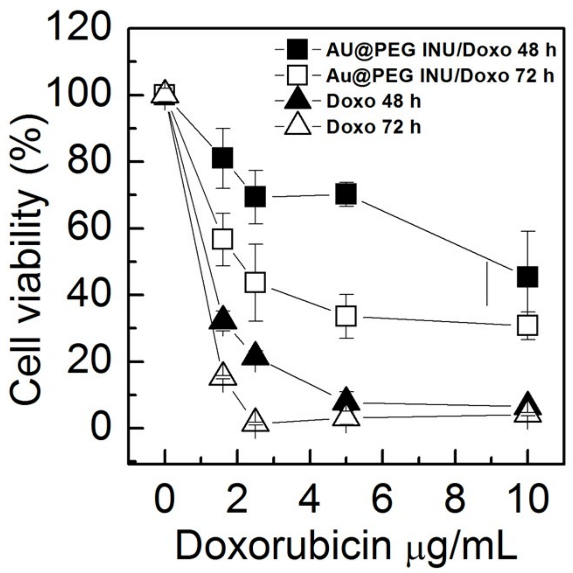

Figure S7. Cell viability (MTT assay) on human lung cancer cells (A549) of doxorubicin hydrochloride and Au@PEG-INU/Doxo. Incubation times were 48h and 72 h.

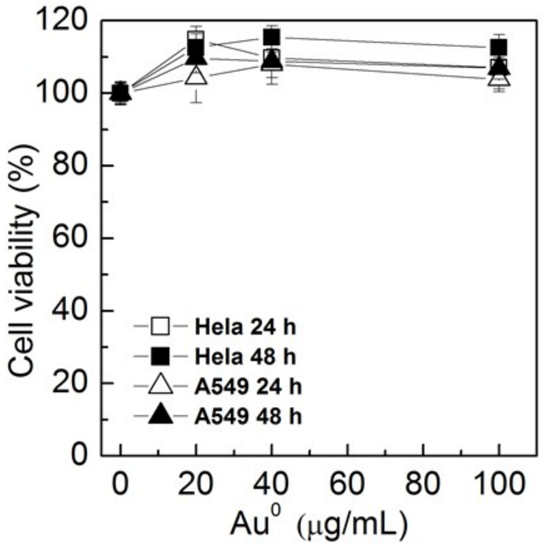

Figure S8. Cell viability of HeLa and A549 cell when exposed to Au@PEG INU NPs for 24 or 48h. Cell viability was measured using the neutral red (NR) assay.

Table S3. IC_{50} of Au@PEG INU/Doxo and free doxorubicin on A549 cells after 48 and 72 h of incubation.

Sample	***IC50 ^{48h} (μM)***	***IC^{72h} (μM)***
Free doxorubicin	*0.97*	*1.12*
Au@PEG INU Doxo	*8.08*	*2.08*

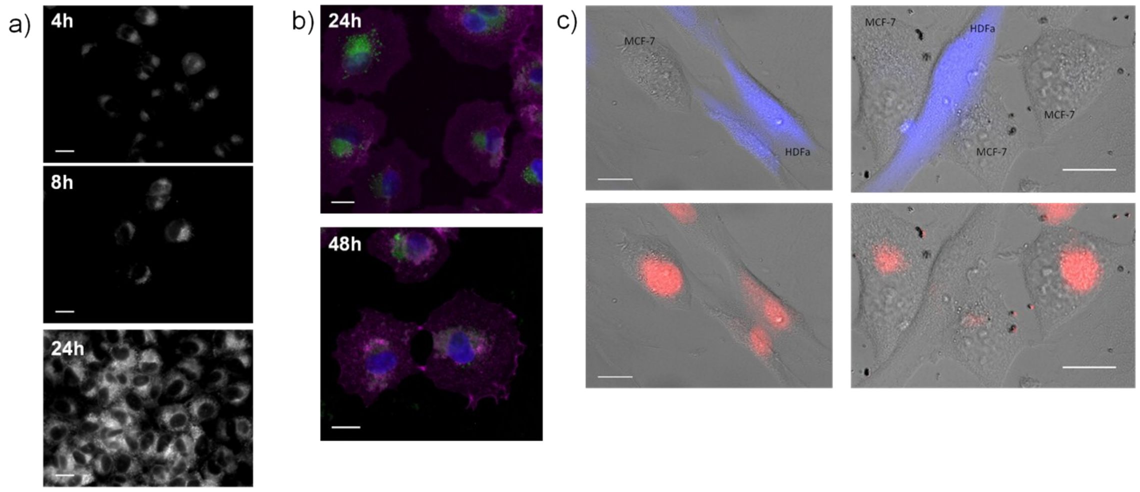

Figure S9. Fluorescence imaging of doxorubicin uptake when delivered via Au@PEG-INU NPs to cancer cells. a) Increasing intracellular presence of doxorubicin in A549 cells at 4h, 8h and 24 h. b) Multistained A549 cells showing doxorubicin (green), nuclear (blue) and

membrane (pink) staining. The decreasing amount of cells is clear when longer incubation times are used. c) Co-culture of MCF-7 breast cancer and HDFa fibroblast cells. HDFa cells were stained with CMF_2HC (blue color). Doxorubicin alone diffuses in both MCF-7 cells and HDFa fibroblast cells (left panel) whereas preferential uptake of Au@PEG INU/Doxo by MCF-7 cells is visible (right panel). CMF_2HC staining alone is shown in the top images, and false colored (red) doxorubicin fluorescence in bottom images. Scale bars are 20 µm.

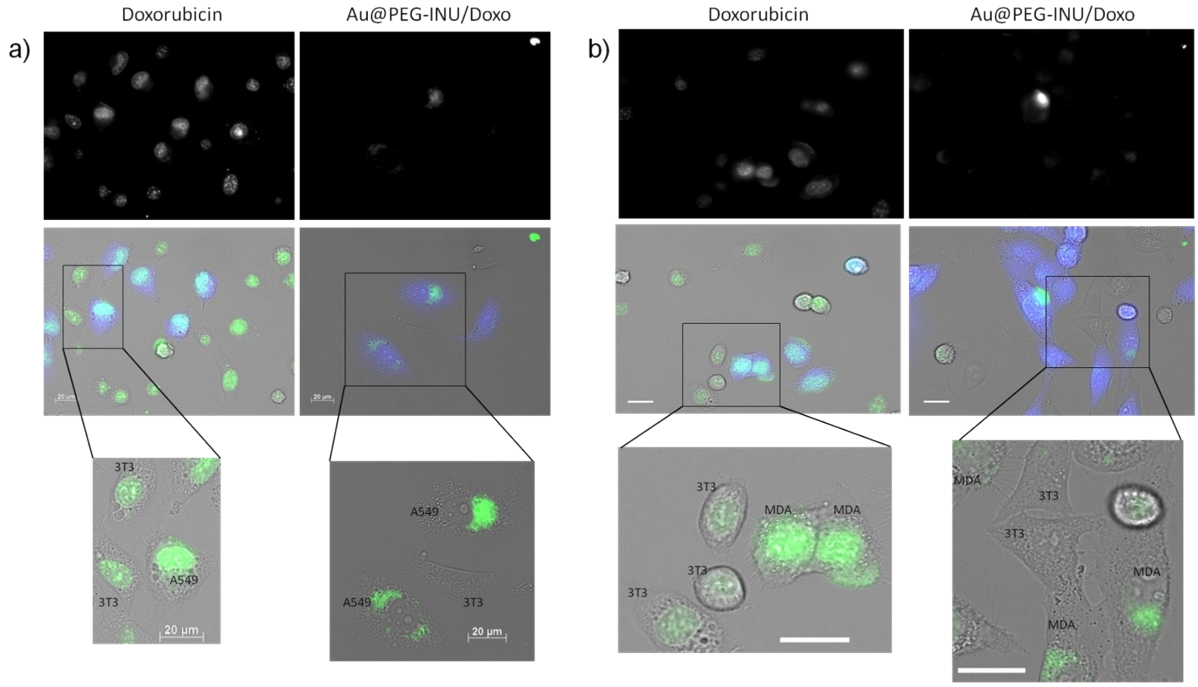

Figure S10. 3T3 fibroblast co-cultures showing preferential uptake of Au@PEG-INU/Doxo nanoparticles by A549 (a) and MDA.MB.435 (b) cancerous cells. A549 broncial adenocarcinoma cells or MDA.MB.435 melanoma cells were pre-stained with CMF_2HC (blue colour) and then co-cultured with 3T3 fibroblasts. Cells were exposed to doxorubicin (left panels) or Au@PEG-INU/Doxo nanoparticles (right panels) for 90 min followed by extensive washing and imaging. Doxorubicin fluorescence alone is shown in the top images and enlarged false colored photos in the bottom images. Scale bars are 20 µm.
